# Liquid slide electrification: advances and open questions

**DOI:** 10.1039/d4sm01289e

**Published:** 2025-01-30

**Authors:** Aaron D. Ratschow, Hans-Jürgen Butt, Steffen Hardt, Stefan A. L. Weber

**Affiliations:** a Institute for Nano- and Microfluidics, TU Darmstadt Peter-Grünberg-Str. 10 64289 Darmstadt Germany; b Max Planck Institute for Polymer Research Ackermannweg 10 55128 Mainz Germany butt@mpip-mainz.mpg.de; c Institute for Photovoltaics, University of Stuttgart Pfaffenwaldring 47 70569 Stuttgart Germany

## Abstract

This review is about drops of a liquid with high dielectric permittivity that slide over a solid surface with high electrical resistivity. A typical situation is a water drop sliding down a tilted hydrophobic plate. It has been realized recently that such drops spontaneously acquire a charge. The opposite charge is deposited behind the drop as a surface charge. Generated electric potentials in the drops can easily reach 1 kV and more. This phenomenon has been termed slide or contact electrification. It is the soft matter analog to triboelectrification, which occurs in solid friction. Slide electrification turned out to be ubiquitous in everyday life and technical applications. It will change our common knowledge of dynamic wetting. Studying slide electrification is complex because the outcome of a wetting experiment depends on the history. For this reason, a series of drops, rather than single drops, are analyzed to gain quantitative understanding. Here, we review the fundamental understanding of slide electrification and its limits. We describe consequences, *e.g.* on drop motion and advancing and receding contact angles and we address open questions.

## Abstract 2

This review is about drops of a liquid with high dielectric permittivity that slide over a solid surface with high electrical resistivity.

## Graphical abstract

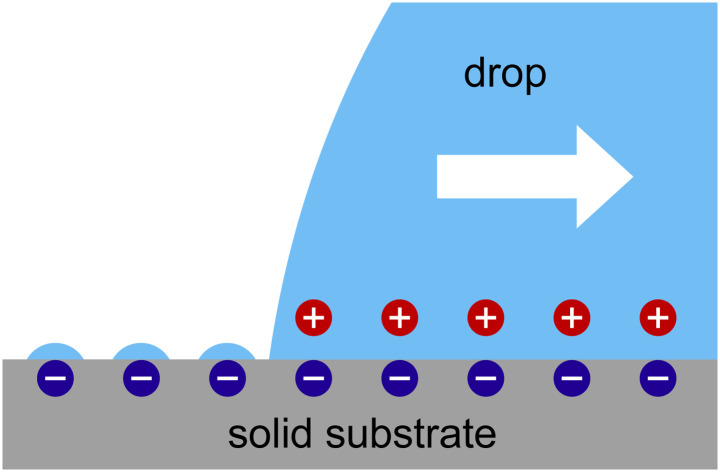

Water drops sliding over insulating, hydrophobic surfaces spontaneously acquire an electric charge.^1–3^ As a result, the solid surfaces become oppositely charged.^4^ Usually, the surface acquires a negative surface charge and the drop becomes positively charged. Similarly, when withdrawing an insulator from a water pool, the solid object is charged, leaving an opposite charge in the water pool.^5–8^ This charge separation at receding contact lines is called slide, dewetting or contact electrification.^1,3^ It is the wet analog to triboelectricity between solids.^9,10^ Electric charging has been observed in micropipetting,^11,12^ spraying,^13,14^ and bouncing of drops.^15,16^ Recent studies suggest that the electric potentials of sliding drops are substantial and can reach a few kV.^17–19^

The phenomenon has been studied for a number of reasons. On the fundamental side, spontaneous charge separation influences the motion of drops^20^ and causes contact-angle hysteresis.^18^ In extreme cases, drops stop moving down tilted plates. With respect to applications, slide electrification has been used to harvest electrical energy.^21–25^ In contrast to conventional generators, slide electrification-based generators do not require moving parts and can be miniaturized for uses in cases like self-powered sensors.^26,27^ The total amount of available energy gain is, however, limited and the efficiency is still low.^28,29^ Slide electrification can also be used to manipulate drop motion; water drops on superhydrophobic surfaces are dragged towards their countercharges.^18^ They could even be made to climb against gravity by imprinting charge gradients onto superhydrophobic surfaces by previous drops.^30^

Here, we provide an overview of the physics behind slide electrification in sessile, moving droplets. We discuss experimental conditions that influence the polarity and magnitude of charge separation as well as the consequences of slide electrification. The aim is to enable estimations of how relevant spontaneous charging is with respect to a specific effect. We point out open questions in the field of slide electrification.

## 1 Charge separation

Charges are separated when a liquid dewets a surface at a moving three-phase contact line. The common model to explain slide electrification starts with the electric double layer, which usually forms spontaneously. The surface charge density at the solid–liquid interface, *σ*_SL_, caused, *e.g.*, by spontaneous adsorption of ions, is compensated by countercharges in a more loosely bound or diffuse layer of countercharges. Together they are called the electric double layer. Current theories describing slide electrification^3,19,31^ and experimental evidence^32^ suggest that the receding three-phase contact line partially strips off these countercharges, leaving behind a certain amount of uncompensated surface charge.^1,4^ An alternative suggestion is that the deposited charges are primarily electrons.^33,34^

To move towards a quantitative understanding of slide electrification, it is instructive to distinguish two effects: first, the formation of the electric double layer, its magnitude and polarity at the solid–liquid interface and a possible change near the three-phase contact line. Second, the charge separation at the rear contact line between the wetted and the de-wetted surface.

### 1.1 The electric double layer

Most solid surfaces in contact with water are electrically charged. Charging is due to the dissociation of surface groups, specific adsorption of ions from the liquid or, on longer timescales, partial dissolution of the surface. The surface charge electrostatically attracts counterions from the liquid into a diffuse layer close to the surface. The characteristic thickness of the diffuse layer is called the Debye length1
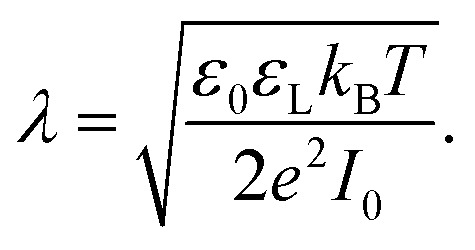


Here, *ε*_0_ is the vacuum permittivity, *ε*_L_ is the liquid's dielectric constant, *k*_B_ is the Boltzmann constant, *T* is the absolute temperature, and *e* is the elementary charge. The Debye length *λ* describes the characteristic thickness of the diffuse layer over an extended flat plate. High salt concentrations, measured using the ionic strength 
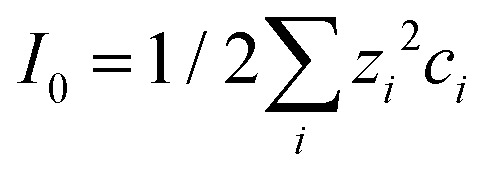
, decrease the Debye length. Here, the sum is over all ionic species, *c*_*i*_ is the bulk concentration of species *i* with valence *z*_*i*_.

The surface charge at the solid surface within the electric double layer is generally given by Grahame's equation. For low surface potentials *ϕ* we can use its linearized form,^35^2
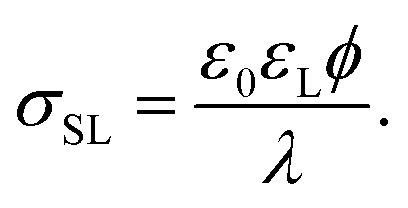


Near the three-phase contact line, the effective thickness of the diffuse layer *λ*_eff_ can deviate from the Debye length of the bulk liquid.

### 1.2 Charge separation at the moving contact line

**Fig. 1:**
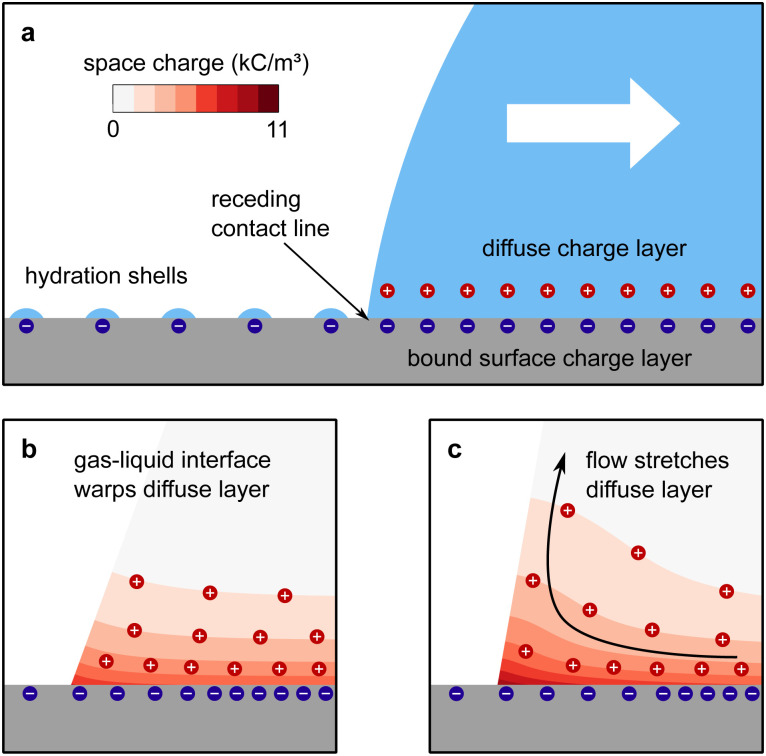
Charge separation process. (a) A part of the bound surface charges from the electric double layer remains on the dewetted surface, possibly with hydration shells. (b) For contact angles <90°, the gas–liquid interface warps the diffuse layer and increases the effective screening length. (c) At high velocities, Pe > 1, the upward flow expands the diffuse layer, increases the effective screening length and decreases the surface charge density near the contact line. Data reproduced from ref. 31.

The mechanism of how charges are separated at the receding contact line is still debated. The most commonly accepted hypothesis is the following: when a surface is dewetted, some of the chemically or physically bound surface charges from the electric double layer remain on the surface, while the countercharge from the diffuse layer remains in the liquid (Fig. 1a). Near the receding contact line, the layer of diffuse charge is warped by the gas–liquid interface.^36^ This deformation can be viewed as a change in the effective screening length. Only for a receding contact angle of *θ* = 90°, the screening length is identical to that far away from the contact line. For *θ* < 90° it is increased because the counterions are forced away from the solid–liquid interface, Fig. 1b. For *θ* > 90° the screening length is smaller than in the bulk. The effect is purely electrostatic and even present for non-moving contact lines. An additional effect is expected for sliding drops. Sliding of the liquid along the surface induces a recirculation flow that points upward near the receding contact line. Advective transport can drive counterions away from the solid surface and increase the effective screening length, Fig. 1c. The strength of the advective transport is measured using the Péclet number, defined as Pe = *Uλ*/*D*, where *U* is the receding contact line velocity and *D* is the ion diffusivity (assumed to be identical for all species). Contact angle and flow effects modify the effective screening length at the receding contact line to^31^3
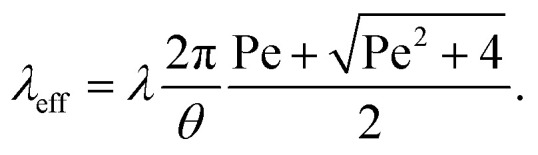


Here, *θ* should be inserted in rad. It refers to the instantaneous dynamic receding contact angle on a smooth substrate. Rough substrates are discussed in Section 4.1.2. For high Péclet numbers Pe ≫ 1, *λ*_eff_ increases proportionally to Pe. For a given surface potential *ϕ*, the surface charge in the electric double layer at the contact line just before dewetting is^31^4
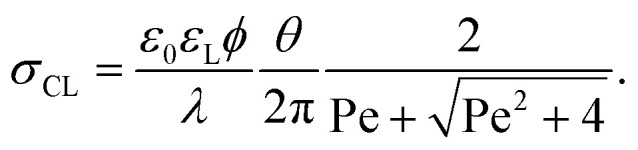
Eqn (4) assumes chemical equilibrium at the solid–liquid interface and a neutral gas–liquid interface. It predicts that charging is strongest on hydrophobic surfaces with high contact angles. This has indeed been observed in experiments.^8,31,37^ At low sliding velocities of the receding contact line, corresponding to Pe < 1, the velocity has no significant influence on charge separation.^31,38^ Even evaporating drops with very low contact line velocities leave behind charges.^5,8,39^ At high velocities, Pe > 1, eqn (4) predicts a decrease in surface charge inversely proportional to Pe. This trend has been measured in sliding drops,^18,26,31^ as well as in the breakup of liquid bridges.^40^

The contact angle and flow effects decrease the surface charge in the liquid directly at the contact line. An unsolved key question is: how much of the surface charge *σ*_CL_ is transferred from the solid–liquid interface to the solid surface right behind the contact line? Experimental observations suggest that during the dewetting, the surface charge that leaves the drop, *σ*_out_, is reduced by a factor *σ*_out_/*σ*_CL_ ≈ 0.5.^31^

Charges are separated although the final state is energetically unfavorable. The electrostatic self-energy of an ion of radius *a* (typically *a* ≈ 0.12 nm) at the interface between two dielectric media with dielectric constants *ε*_S_ and *ε*_L_ is *U*_*i*_ = *e*^2^/[4π*ε*_0_(*ε*_S_ + *ε*_L_)*a*]. With the dielectric constants *ε*_S_ = 5 for the solid and *ε*_L_ = 80 for water, self-charging requires an energy of 5*k*_B_*T*. If water is replaced by air with permittivity *ε*_G_ = 1, the corresponding energy is 78*k*_B_*T*. Thermal energy is sufficient for charge separation in water but not in air.

To explain why a significant number of surface charges are transferred from the wet to the dry region, even though the above estimate suggests that this is energetically unfavorable, we assume that the surface charges that stay on the dewetted surface retain hydration shells.^3,8,31^ Near the contact line, humidity is still close to saturation. For this reason, an adsorbed water layer will remain on the surface. These high energies suggest that charge separation at the receding contact line is a non-equilibrium process.

According to the idea that the surface charge *σ*_out_ is a leftover from the electric double layer, it is reasonable to describe the overall charge separation process with a fraction *α* of the charges at the solid–liquid interface, which remains on the surface:

5 *σ*_out_ = *ασ*_SL_.

The effective charge transfer coefficient *α* (0 ≤ *α* ≤ 1) summarizes the effects of the contact angle, Péclet number and effects on the atomistic scale, such as hydration shells.

## 2 Charge accumulation in moving drops

### 2.1 Single drops

Charge separation at receding contact lines is not unique to drops. It occurs in many dynamic wetting contexts,^8,11–13,40^ for example when withdrawing a plate from a liquid pool. What sets drops apart is that they are electrically isolated volumes of liquid. They can accumulate a defined charge *Q*. How does the drop charge evolve, when a drop slides over a surface?

As a drop slides on a substrate, it deposits surface charges and acquires an opposite drop charge *Q* that increases and eventually saturates with the slide distance. The change in *Q* due to charge separation along the sliding path *x* follows^3,19^

6 d*Q*(*x*) = −*ασ*_SL_(*x*)*w*d*x*.

Here, *w* is the drop width and *α* is the effective charge transfer coefficient, as introduced in eqn (5).

As the drop charges and deposits surface charges,^20^ it develops a drop potential *U*_d_. Potential and charge are linked *via* the drop capacitance *C*_d_, according to *U*_d_ = *Q*/*C*_d_. In many experiments, there is a metallic electrode underneath the dielectric substrate of thickness *d*. Neglecting rim effects (*d* ≪ *w*) and assuming *λ* ≪ *d*, the drop capacitance is7
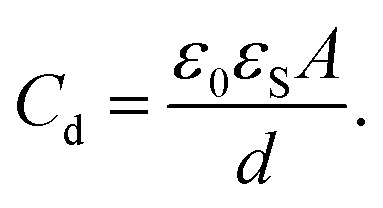


Here, *A* is the contact area of the drop and *ε*_S_ is the dielectric constant of the substrate.

When the drop is charged, an electric field *U*_d_/*d* exists in the dielectric substrate. Grahame's equation needs to be replaced by the Gauss law at the solid–liquid interface. Assuming moderate surface potentials, *ϕ* < *k*_B_*T*/*e*, it reads^19,31^8
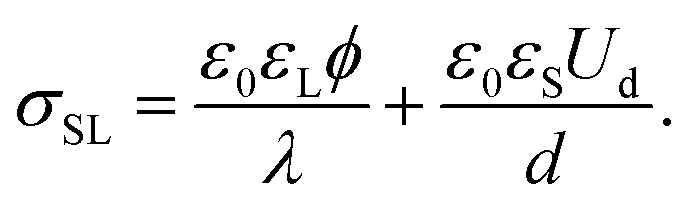


Experiments suggest that the surface potential *ϕ* does not significantly vary with the drop voltage *U*_d_.^19^Eqn (6)–(8) then predict an exponential charging on a saturation sliding length scale9
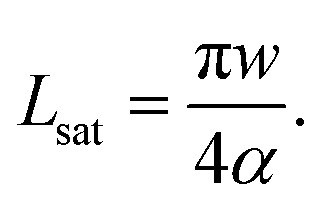


Here, we approximated *A* ≈ π*w*^2^/4. Experimentally, *L*_sat_ is usually of the order of 1 cm.^3,19,32^

The drop charge and drop voltage saturate when d*Q* = 0. Following eqn (8), the saturation potential of the drop is^19^10
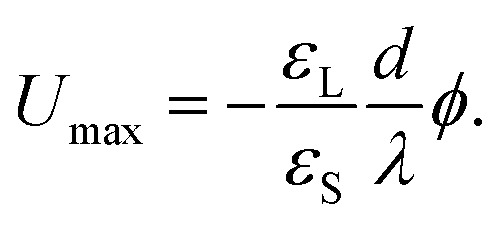


With typical surface potentials *ϕ* ≈ −40 mV and substrate thicknesses *d* ≈ 1 mm, drops saturate at ≈1 kV.^17–19^ Notably, the saturation charge, *Q*_max_ = *C*_d_*U*_max_ = *Aε*_0_*ε*_L_*ϕ*/*λ* only depends on the liquid and the surface potential.

**Fig. 2:**
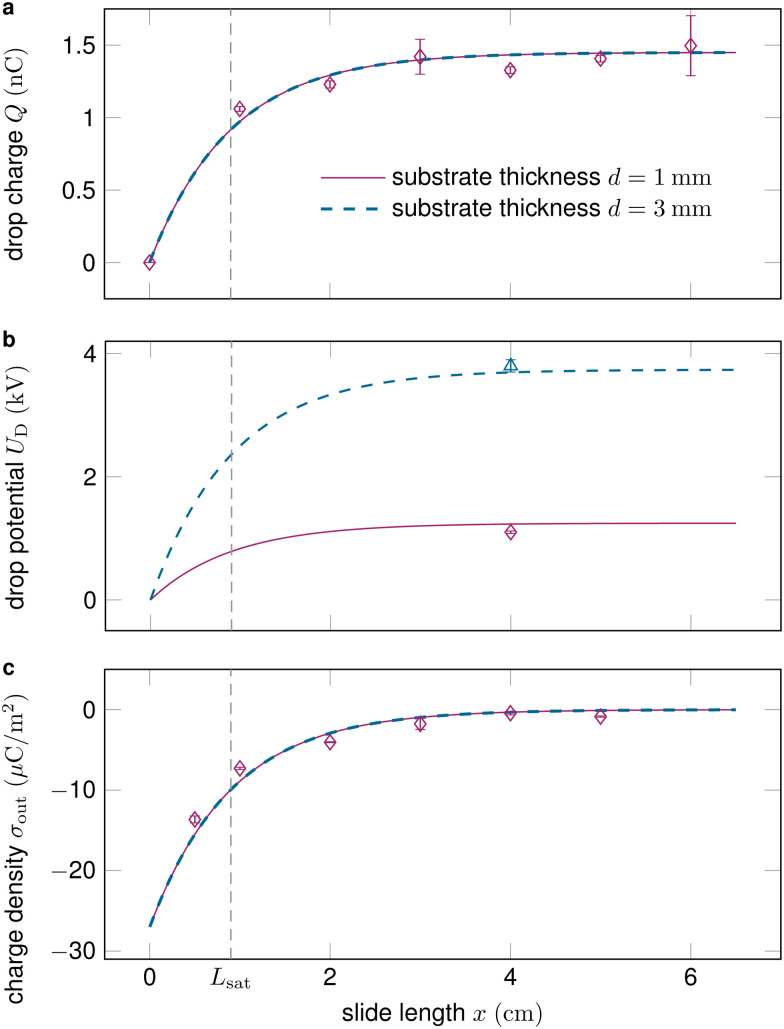
(a) The drop charge along the slide length typically saturates after a couple of centimeters and is essentially independent of the substrate thickness. (b) Drops sliding on thicker substrates obtain substantially higher voltages, see eqn (10). (c) The surface charge density after the drop *σ*_out_ decreases along the slide path and vanishes at saturation. Data reproduced from ref. 19, 41.

Fig. 2 shows how the drop charge saturates on the length scale *L*_sat_ as the deposited surface charge decays. While the drop charge *Q* (Fig. 2a) and surface charge density *σ*_out_ (Fig. 2c) are essentially independent of the substrate thickness *d*, the drop potential *U*_D_ increases on thicker substrates (Fig. 2b).

### 2.2 Charge neutralization

How long do surface charges remain on a surface? Above, we assumed that the substrate is insulating. Real materials have a finite resistivity *ρ*_S_ (in Ω m or V m A^−1^). Consequently, a charged drop will eventually discharge, or the surface charges left behind by a drop will eventually disappear. For a substrate of constant thickness with a back electrode, the characteristic discharge time is *τ* = RC = *ε*_0_*ε*_S_*ρ*_S_. It is independent of the substrate thickness, assuming a homogeneous substrate material.

For many materials, the characteristic discharge time is much longer than the sliding time of a drop and sometimes even longer than typical evaporation times. Soda lime glass discharges within seconds. On quartz glass, the charges are stable over days.^32^ We give the resistivity and dielectric constant of typical materials in Table 1.

**Table 1:** Dielectric constant *ε*_S_, resistivity *ρ*_e_, and dielectric strength *E*_bt_ of common materials in slide electrification. Typically ranges are given because the values depend on the specific preparation, frequency, duration of exposition and thickness

Material	*ε*_S_	*ρ*_e_ (Ω m)	*E*_bt_ (MV m^−1^)
Polyethylene (PE)	2.2–2.4	10^13^–10^16^	19–160
Polyethylene terephthalate (PET)	3.0–3.4	10^14^–10^16^	17–25
Polymethylmethacrylate (PMMA)	3.8–4.2	2 × 10^13^–10^15^	15–60
Polypropylene (PP)	2.2–2.4	10^13^–10^15^	30–200
Polystyrene (PS)	2.3–2.5	10^15^–10^16^	20–200
Polyvinyl chloride (PVC)	3.0–8.0	10^12^–10^14^	12–72
Nylon	3.5–5.0	10^10^–10^15^	18–30
Polytetrafluoroethylene (PTFE)	2.0–2.1	10^16^–10^18^	20–285
Polycarbonate (PC)	2.7–3.2	10^14^–10^15^	12–67
Polydimethylsiloxane (PDMS)	2.3–2.7	10^13^–10^16^	22–250
Silicone oil	2.3–2.8	10^12^–10^13^	10–15
Quartz (SiO_2_)	4.5	10^14^–10^18^	25–100
Silicon dioxide amorphous	3.8–3.9	10^16^–10^18^	400–670
Borosilicate glass	4.1–5.1	10^15^–10^17^	20–40

In addition to neutralization through the substrate, surface charges may be discharged by ions in air or a possible conductivity of the surface. Ions in air originate, *e.g.*, from cosmic radiation or radioactive sources.^42^ Humid air may also contain H_3_O^+^ and OH^−^ ions.^9^ Under atmospheric conditions, discharge through ions in air is generally negligible. Only at a humidity above 80%, drop charging tends to decrease,^43^ indicating an increase in surface- or air conductivity. Even when increasing the ion concentration with ionizing air blowers, neutralization through air requires several seconds.^32^

### 2.3 Drop sequences

In many practical applications, multiple drops successively slide down the same surface. In addition, measuring the charge of a series of drops has turned out to be a good way to characterize drop charging.^3^ Unless the time between drops Δ*t* is much longer than the characteristic discharge time of the surface *τ*, a drop will encounter charges left by a previous drop *σ*_in_. The drop absorbs these charges at its advancing contact line. Then, it deposits new surface charges *σ*_out_ at its receding contact line, see Section 1. During the time between drops, the surface charges partially dissipate, so that^3,19^

**Fig. 3:**
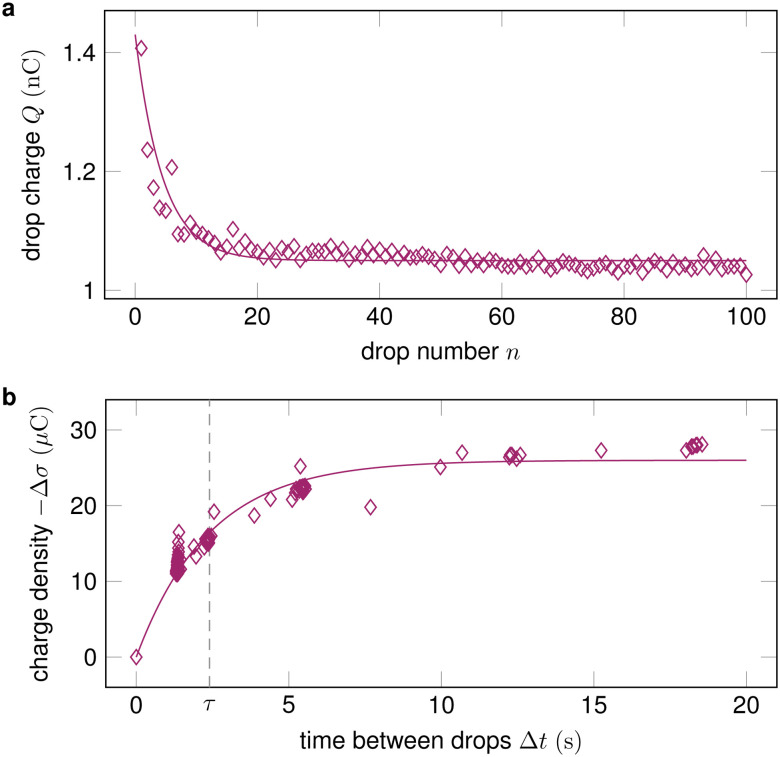
(a) The drop charge at a fixed position *x* along the slide path decreases and then saturates with increasing drop number. Data reproduced from ref. 19; (b) By varying the time between drops Δ*t* and measuring the change in surface charge density Δ*σ*, the characteristic discharge time *τ* can be quantified. Data reproduced from ref. 32.

11 *σ*_in,*n*_ = *σ*_out,*n*−1_ exp(−Δ*t*/*τ*),

where *n* is the drop number. The discharge time of the surface *τ* is discussed in Section 4.1.3. After a number of successive drops, a steady state is reached where, at each position *x*, the increment in the surface charge from a passing drop fulfills Δ*σ* = *σ*_out_ − *σ*_in_ = *σ*_out_[1 − exp(−Δ*t*/*τ*)] (Fig. 3).^32^ Consequently, the saturation sliding length scale increases to *L*_sat,*n*→∞_ = π*w*/{4*α*[1 − exp(−Δ*t*/*τ*)]}. Drops will still continue to deposit charges until they reach saturation (eqn (10)). However, for short Δ*t*, the total length of the surface may not be sufficient for saturation.

## 3 Consequences of slide electrification

**Fig. 4:**
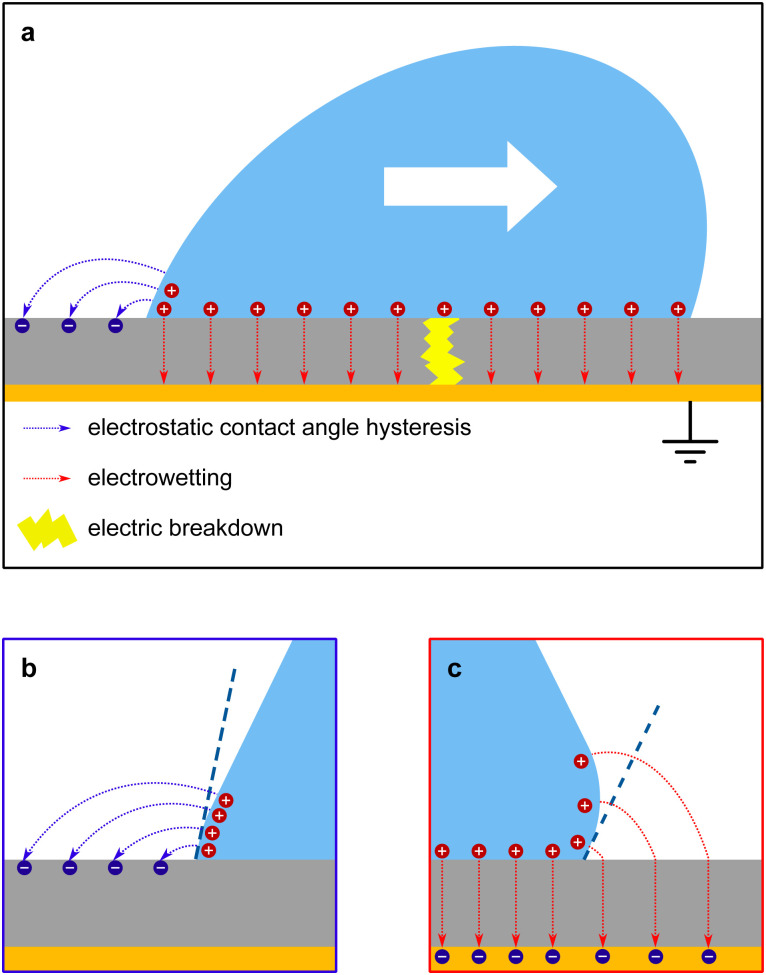
(a) A sliding drop affected by electrostatic contact angle hysteresis, electrowetting, and electric breakdown. (b) and (c) Details of the electric fields causing Maxwell stress on the drop in the case of electrostatic contact angle hysteresis (b) and electrowetting (c).

Important consequences of slide electrification are retardation of drop sliding, decreasing advancing and receding contact angles and possible electric breakdown (Fig. 4a).

For the subsequent analysis, we consider a planar substrate of thickness *d*, which could be a polymer, glass, or SiO_2_. This substrate is naturally hydrophobic (*e.g.*, polystyrene or polytetrafluoroethylene, PTFE) or is coated with a layer (*e.g.*, with fluorinated silane) to adjust the contact angles. We assume that such a coating has negligible thickness. At the bottom side, the substrate is coated with a metal. The metal is grounded.

### 3.1 Contact angle hysteresis

Static contact angle hysteresis is the difference between the static advancing *θ*_a_ and receding contact angles *θ*_r_ of a drop, CAH = *θ*_a_ − *θ*_r_.^44,45^ On a tilted surface, it determines whether a drop sticks to or rolls off a surface. The net capillary force acting on a drop is given by the Kawasaki–Furmidge equation^46–49^

12 *F* = *wγ*_L_*k*(cos *θ*_r_ − cos *θ*_a_).

Here, *γ*_L_ is the liquid surface tension and *k* is a dimensionless geometric factor ≈1. A drop slides off a tilted surface once the gravitational force, *mg* sin *α*, exceeds the capillary force (*m* is the mass of drop, *g* the gravitational acceleration, and *α* is the tilt angle). Contact angle hysteresis has been investigated for decades and is traced back to various factors, including heterogeneity,^50–52^ roughness,^53–55^ surface defects,^56^ surface deformation,^57^ molecular kinetics at the contact line,^58,59^ and surface adaptation.^60^

Recently, it was revealed that the electrostatic interaction between a drop and deposited surface charges from slide electrification causes a substantial force on sliding drops.^20^ This mainly manifests in the deformation of the drop. It decreases the contact angle at the receding contact line, where charges are deposited. The advancing contact angle only decreases if the surface at the advancing contact line carries charge deposited by previous drops.^18^ Thus, slide electrification causes a fundamental contribution to contact-angle hysteresis. Microscopically, Maxwell stress near the contact line causes the deformation (Fig. 4b). Macroscopically, the effect can be attributed to an effective increase in the solid surface energy caused by the mutual repulsion of the charges on the surface. Additionally, the surface charges electrostatically interact with countercharges in the receding liquid. Thus, the energy required to dewet the surface increases compared to a case without charge separation. The change in solid surface energy at the receding side of the drop scales like^18^13
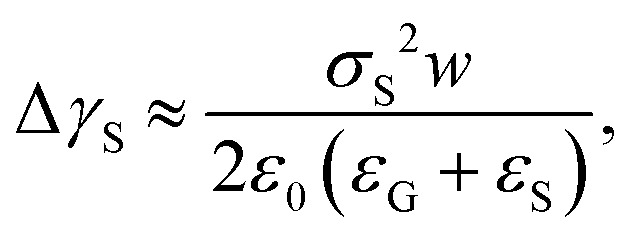
where *σ*_S_ is the surface charge density on the substrate next to the contact line and *ε*_G_ and *ε*_S_ are the dielectric constants of the surrounding gas and the substrate, respectively. The scaling in eqn (13) has been derived from the energy required to deposit additional charges on a charged circular area of diameter *w*. The effect increases with the size of the charged area, and correspondingly, the drop width.^18^ The change in the contact angle caused by the surface charge is^18^14
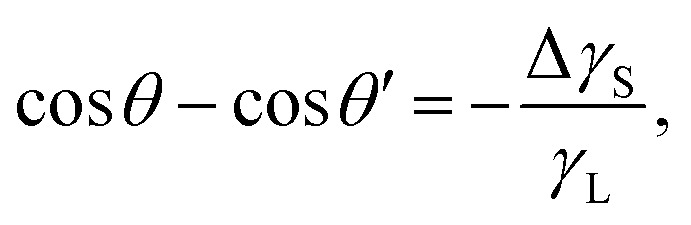
where *θ* and *θ*′ are the contact angles without and with the electrostatic contribution, respectively. This effect occurs even when the liquid is grounded. The conductive liquid surface then carries induced countercharges. The electrostatic contribution to contact angle hysteresis can be of the order of 10°.^18^

Long-range electrostatic interactions between the drop and the surface charge can further slow down drop motion.^20^

Because charges separate at receding contact lines, contact angles reduce after the first wetting–dewetting cycle^61^ and can only be recovered after times much longer than the surface discharge time *τ*, when the surface charge has been neutralized. The interaction of drops with deposited surface charges can even stop moving drops.^20^ Drops can spontaneously move along surface charge gradients, even against gravity.^30^ Consequently, subsequent sliding drops tend to align with the trajectories of previous drops.

### 3.2 Electrowetting

Electrowetting on dielectric (EWOD) is the modification of the wetting properties of a surface with a dielectric coating by an electric field. A drop is placed on a dielectric layer covering a flat electrode. When a voltage Δ*U* is applied between the drop and the electrode, the contact angle decreases. The effect is again caused by the electrostatic Maxwell stress on the liquid surface (Fig. 4c). It can be modelled as a decrease in the effective energy, or interfacial tension, of the solid–liquid interface caused by the diffuse layer charges. The decrease in surface energy is calculated as follows^62^15
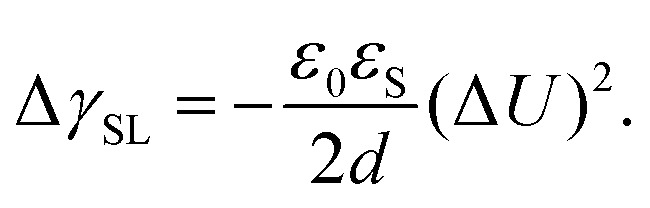


This assumes 
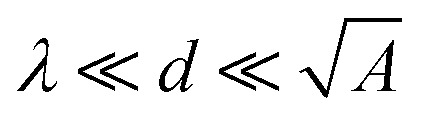
, where *A* is the wetted area. In the case of spontaneous charging by slide electrification, the applied potential, Δ*U*, has to be replaced by the drop potential, *U*_d_. The Young–Lippmann equation gives the contact angle change,^63,64^16
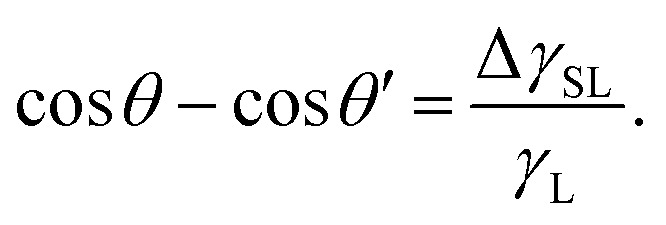


Again, *θ* and *θ*′ are the contact angles without and with an applied voltage.

EWOD applications typically use substrate thicknesses around 1–10 μm and voltages of ≈100 V.^61^ In slide electrification, substrate thicknesses are of the order of 1 mm and drops acquire kilovolt potentials. These conditions are sufficient for electrowetting and indeed, contact angle reductions in aqueous drops of the order of 10–20° have been reported.^18^ Electrowetting requires a potential difference between the drop and the electrode beneath the substrate. It does not occur when the liquid is grounded or connected to a large reservoir.

Contact angle reduction by electrowetting is symmetric around the whole contact line and affects receding and advancing contact angles. It increases the wetted area but otherwise has little effect on drop friction or contact angle hysteresis.

### 3.3 Electric breakdown

When a drop is fully charged and has reached saturation, the electric field strength in the dielectric substrate is *U*_max_/*d* = −*ε*_L_*ϕ*/(*ε*_S_*λ*) (eqn (10)). It can be quite high. For a water drop with 1 mM salt (*λ* = 10 nm) on quartz and assuming a surface potential *ϕ* = −40 mV, the field strength reaches 70 V μm^−1^. This value is lower than, *e.g.*, the dielectric strength of amorphous SiO_2_, which is *E*_bt_ = 470–1000 V μm^−1^, and breakdown is unlikely. Many other substrate materials, however, may show a breakdown, see Table 1.

Moreover, the maximum electric field strength increases with decreasing Debye length. In this way, increasing the salt concentration can lead to an increase of the electric field across the substrate. We can calculate the critical Debye length, *λ*_c_, where breakdown will likely set in,17
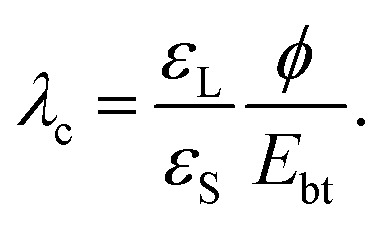


This assumes that the surface potential *ϕ* is roughly independent of the Debye length. For many materials electric breakdown and possible degradation of the sample is a realistic scenario. The higher the salt concentration, the higher the chance for electric breakdown when drops slide on the surface. Damage due to electric breakdown commonly occurs in semiconductor manufacturing during the rinsing of wafers.^65–67^ In nanoelectronics, the nC charges of sliding drops can further cause damage through resistive heating.

In Table 1 we report *ε*_S_, *ρ*, and *E*_bt_, measured for thick samples. For thin films, these values generally depend on the thickness. Zhao & Liu^68^ recommend to describe the dielectric strength empirically by *E*_bt_ = *E*_1_/*d*^*a*^. Here, the exponent 0 < *a* ≤ 1 depends on the time scale, temperature, electric field uniformity and thickness.

### 3.4 Gas discharge and breakup of drops

The liquid close to the contact line can be represented as a wedge.^69^ For the model problem of a wedge formed by an isopotential and a charged surface, the electric field has a singularity at the sharp corner representing the contact line.^70^ In reality, there must exist a mechanism cutting off the singularity.^18^ Still, large electric field strengths are expected close to the contact line that could exceed the threshold field strength for gas discharges. Corresponding discharges near the contact line of sessile drops have been observed in high electric fields,^71^ and in electrowetting.^72^ More than 300 years ago, J. Picard and J. I. Bernoulli reported that light is emitted from moving mercury. The generated electric field is strong enough to cause glow discharge along the contact line.^73^ To the best of our knowledge, this has not yet been reported in sliding aqueous drops. However, the electric field configurations present in such experiments suggest that gas discharges may also occur there.

Charges in a drop repel each other and can even overcome the surface tension. The drop then disintegrates and ejects smaller charged droplets. The maximum charge that a spherical drop of radius *R* in a vacuum can carry before becoming unstable is given by 
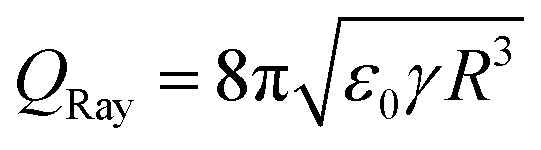
, which is known as the Rayleigh limit.^74^ For example, for water and drops of 2 mm radius this limiting drop charge is 1.8 nC. A similar instability can also be expected for drops on surfaces that have acquired a large charge, but is yet to be confirmed in slide electrification experiments. In electrowetting experiments, highly charged drops have been observed to disintegrate and release small drops at their contact line.^61,62^

## 4 Influence of drop and substrate properties

### 4.1 Substrate

#### 4.1.1 Chemical composition of the surface

Slide electrification of drops has so far only been reported for hydrophobic surfaces with static receding contact angles above ≈70°. The reason is still being debated.^75^ Charging increases with an increasing receding contact angle, as explained by eqn (4).^8,31,37^ The lack of data for slide electrification on hydrophilic surfaces could also be explained by the fact that drops with low contact angles do not slide or easily disintegrate when sliding.

The most common explanation for interfacial charges is an enrichment of hydroxyl ions at the interface.^2,76^ Alternative hypotheses see the origin of interfacial charge in the asymmetry of the hydrogen-bond network,^77^ adsorption of bicarbonate/carbonate ions^78,79^ or the transfer of electrons from water to the polymer substrate.^80^

Generally, fluorinated surfaces such as Teflon or glass coated with fluorinated silanes lead to the highest separation of charges. It is not yet clear if this is only due to their high contact angles or if the especially high electronegativity of fluorine plays an additional role. Charge separation also happens on polymers such as polyamides, polyethylene, poly(vinyl chloride), polydimethylsiloxane (PDMS, silicone), polyethylene terephthalate (PET), or poly(methyl methacrylate).^8,20,22,32,81^ Furthermore, thin organic layers such as monolayers of octadecyl-trichlorosilane (OTS) on glass or SiO_2_ are negatively charged by sliding water drops.^37^ Such thin layers can be penetrated by water molecules. Thus, the specific chemical composition of the substrate influences charging.^82^ Dissociable groups on the surface also have an influence. For example, glass coated with amine-terminated silanes leads to negatively charged drops and a positive surface charge.^83^

#### 4.1.2 Surface roughness

The arguments of Section 1 assume a smooth surface. Yet, real surfaces are never perfectly smooth. On a rough surface the contact line can get pinned at local defects (local elevations)^84^ and propagate in an erratic and discontinuous manner.^85^ Reconciling theoretical models for contact-line propagation on rough surfaces with models for charge separation will pose significant challenges for future research. Empirically, a gradual decrease in charging with increasing roughness ≥ 10 nm has been observed.^82^

A special case occurs for superhydrophobic surfaces. When hydrophobic surfaces are sufficiently rough or even microstructured, a wetting transition from the Wenzel to the Cassie–Baxter state^84^ occurs. This effect decreases the solid–liquid contact area, which reduces charging due to slide electrification.^86–88^

#### 4.1.3 Substrate material and thickness

Static contact angles are determined by the chemical composition of the top few nanometers of a surface and short-range surface forces. Electrostatic forces are long range. For this reason, the substrate material plays an important role in slide electrification.

On very thin substrates with a thickness *d* of a few nm or a few 10 nm with a conductive material underneath, charging is usually suppressed. It seems that inhomogeneities in such thin layers lead to fast discharging of drops. Furthermore, the electric field emanating from charges that are more than ≈*d* away from the contact line is screened by the underlying grounded metal. For substrates thicker than 10 nm, the substrate material's RC-time, *τ* = *ε*_0_*ε*_S_*ρ*_S_, dictates the longevity of drop and surface charges, as discussed in Section 2.2. On substrates with low dielectric strength, see Table 1, electric breakdown can prevent charge saturation.

The substrate's dielectric constant has a big influence on slide electrification. Although *ε*_S_ does not influence charge separation and accumulation so much, it changes the drop potential and drop motion. The saturation potential decreases ∝ *ε*_S_^−1^ due to an increase in drop capacitance. As a result, the electric field strength in the substrate decreases with increasing dielectric permittivity. On substrates with high dielectric permittivity, electrostatic contact angle hysteresis also decreases. The higher permittivity decreases the effective field emitted by the surface charges (Fig. 4c), and the effective increase in interfacial tension of the solid, eqn (13).

The substrate thickness influences the saturation potential (eqn (10)): *U*_max_ increases linearly with the substrate thickness, according to eqn (7). Due to the long range of electrostatic forces, conducting parts of an experimental setup which are millimeters away can also influence the drop potential and drop movement.

Substrate treatment and preparation can play a substantial role. This is particularly true for glass or SiO_2_ coated with monolayers. If, *e.g.*, plasma cleaning is used in a specific step, long-term stable charge can be deposited.^89^ This effect is most likely caused by subsurface deposition of long-living charges during the plasma process.

### 4.2 Liquid

#### 4.2.1 Drop size and shape

A drop on a surface assumes different shapes, depending on how its characteristic size *V*^1/3^ compares to the capillary length 
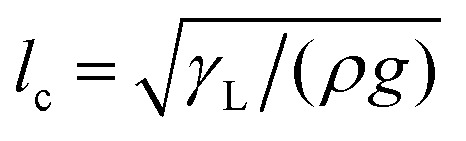
. Here, *V* is the drop volume, and *ρ* is the liquid's mass density. For *V*^1/3^ < *l*_c_, the drop takes the shape of roughly a spherical cap on a homogeneous surface. By contrast, for *V*^1/3^ ≫ *l*_c_, it appears as a liquid puddle.

Larger drops tend to experience increased electrostatic contact angle hysteresis^18^ and stronger electrostatic forces, hindering their motion. The increase in effective solid interfacial tension is proportional to the drop width *w* ∝ *V*^1/3^ according to eqn (13). This trend has also been observed experimentally.^18^ The corresponding additional electrostatic force increases quadratically with the drop width, as predicted by eqn (12) and (13). In contrast, the gravitational force on a sliding drop is proportional to *V*.

Even though smaller drops experience less contact angle hysteresis, they have an increased tendency to stick due to electrostatic effects. Their gravitational force ∝*V* is smaller than the electrostatic force ∝*V*^2/3^ hindering their movement. Smaller drops also saturate earlier, as the saturation sliding length, eqn (9), is proportional to *w*. At saturation, they carry substantially less energy than larger drops since the drop capacitance, eqn (7), is proportional to the drop contact area *A* ≈ π*w*^2^.

Charge separation is not restricted to liquid drops. It is also observed when plates are pulled out from a liquid pool.^8,90^ Electrostatic forces substantially slow down the breakup of liquid bridges on surfaces.^40^ They also slow down the rebound of impacting water drops on insulating surfaces.^16^ This suggests that this phenomenon occurs for a broad variety of liquid configurations on surfaces.

#### 4.2.2 Liquid dielectric constant

Only polar liquids lead to charging. A higher liquid permittivity leads to two competing effects. The Debye length scales *λ* ∝ *ε*_L_^1/2^ (eqn (1)), which would lead to a lower surface charge in the electric double layer. This is counteracted by the proportionality of *σ*_SL_ ∝ *ε*_L_, eqn (2), and ultimately yields higher charging and a higher saturation charge *Q*_max_. In contrast, in liquids of low dielectric permittivity, the dissociation of normal inorganic salts into ions and the formation of an electric double layer are largely suppressed.^91^ In practice, charge separation is negligible for *ε* ≤ 20. Sliding mercury drops, which are conductive, show strong charging.^73^ In liquids with a permittivity below 20, the electrostatic self-energy, or Born energy, is too high. Ions start to dissolve only when the ionic radius increases above 1 nm.

#### 4.2.3 pH

It is well established that the solid–liquid interfacial charge is negative at neutral pH and becomes more negative with increasing pH. Most hydrophobic materials show an isoelectric point around pH = 3.^92–96^ For water on hydrophobic surfaces (hydrocarbons or fluorinated hydrocarbons), Sosa *et al.*^43,76^ observed that charging increased up to pH = 10 on PTFE. In contrast, Nie *et al.*^97^ reported a maximum charge separation on PTFE around pH = 7. For higher pH, they observed a decreasing effect. For water drops ejected from a PTFE capillary, Artemov^98^ also found a maximum at neutral pH. Sbeih *et al.*^99^ found maximum charging around pH = 7 for PFOTS and PDMS surfaces. Under acidic conditions, when the pH is below the isoelectric point or point of zero charge of the surface, even charge reversal has been observed, where the drops became negatively charged while the surface remained positively charged.^5,43,97^

Generally, experiments with varying pH values should be performed at a constant ionic strength. This prevents additional effects due to a change in the Debye length.

#### 4.2.4 Salt concentration

**Fig. 5:**
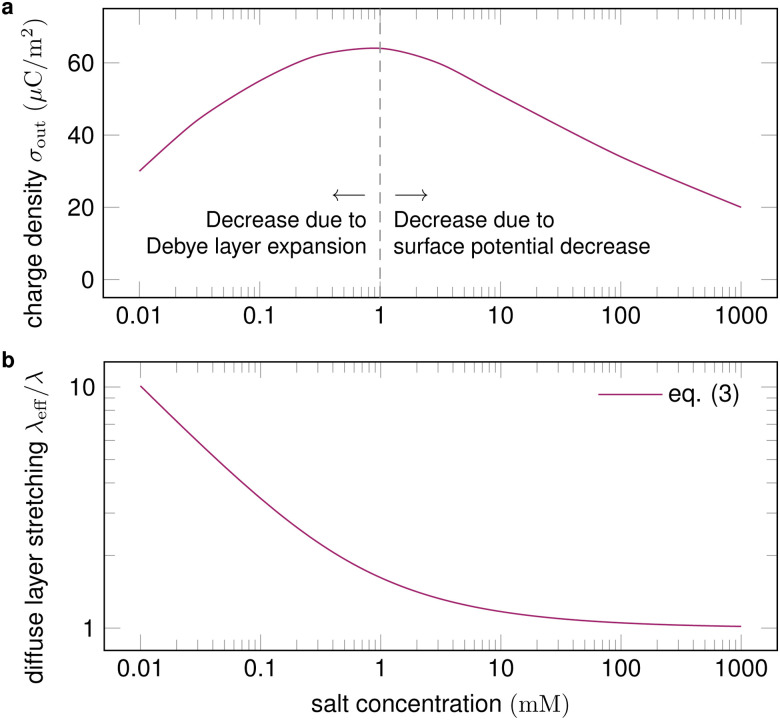
(a) Surface charge density as a function of salt concentration (exemplary values), with proposed physical mechanisms for the trends. Charge separation first increases with salt concentration and then decreases. (b) For a given velocity of the order of 10 cm s^−1^, the expansion of the diffuse layer is strongest for low salt concentrations and diminishes at high concentrations. Trend line calculated with eqn (3).

Slide electrification is a universal and robust effect on dielectric surfaces. It does not only occur in distilled water. When adding salt, charging has been reported to first increase up to a monovalent salt concentration of 0.01–1 mM and then decrease (Fig. 5a).^18,99^ The salt concentration generally influences the Debye length, the interfacial charge density at the liquid–solid interface, and the surface potential of the solid–liquid interface.^96,100,101^

A higher salt concentration decreases the Debye length according to *λ* ∝ *I*_0_^−1/2^, eqn (1). The Debye length determines how much the fluid flow can expand the diffuse layer (Fig. 1c). At a given flow velocity, thinner diffuse layers expand less. Thus, adding salt first decreases and then suppresses the expansion of the Debye layer, measured by the Péclet number, as illustrated in Fig. 5b. The expansion decreases charge separation. This explains the increase of charge separation up to 0.01–1 mM salt.

Variation of the salt concentration also brings along a surface potential variation,^96^ which makes the interpretation of corresponding experiments difficult. Indeed, the experimental data reported in ref. 99 do not show any clear trend of how charge separation depends on the salt concentration at high concentrations.

The relationship between the surface potential and the salt concentration is an open question in the field of physical chemistry. Very high salt concentrations of the order of 1 M lead to reduced charging of the solid–liquid interface.^96,102^ Correspondingly, little charging of drops is observed at salt concentrations above ≈0.4 M.^18,43,97^

The specific salt in the electrolyte influences slide electrification.^18^ This is most likely due to specific adsorption of ions at the solid–liquid interface. Experiments show that the influence of the specific salt is moderate at low concentrations ≈0.01 mM and increases with the salt concentration.^99^ Yet, no general trend can be deduced.

#### 4.2.5 Liquid viscosity

The liquid viscosity *μ* does not directly influence charge separation or charge accumulation. The Debye length *λ* does not depend on *μ*. However, the liquid viscosity influences the Péclet number Pe = *Uλ*/*D*. Assuming spherical ions with radius *r*, the Stokes–Einstein relation leads to a diffusion coefficient *D* = *k*_B_*T*/(6π*μr*).^103,104^ Higher viscosity decreases the ion diffusivity and would thus increase the Péclet number.

Often, dewetting processes get slowed down with increasing viscosity. Eqn (3) predicts that the stretching of the diffuse layer increases with Pe, which means that the surface charge near the contact line *σ*_CL_ decreases less at higher viscosities.

A special case exists when the counterion cloud mainly contains protons/hydronium ions. Then, the diffusive transport of counterions occurs *via* the Grotthuss mechanism,^105,106^ to which the Stokes–Einstein relation does not apply. The resulting proton transport is expected to be approximately as fast as in pure water. Here, the viscosity decreases the velocity *U* and thus reduces the Péclet number, which leads to higher charging. This has been observed for water–glycerol mixtures,^40^ where ion transport is governed by the Grotthus mechanism.^107,108^ The surface tension, dielectric constant, and Debye length vary by a factor <2 between pure water and pure glycerol.^109–111^ Only the viscosity varies over three orders of magnitude with varying glycerol contents.

### 4.3 Solid–liquid interface

Often, a Stern layer is found at solid surfaces in contact with water.^112,113^ In its simplest version, this layer consists of ions that are not completely hydrated and are adsorbed to the surface. Therefore, what remains on the surface after dewetting may not be the bare surface charge, but the surface covered with a Stern layer. In some cases, ions in the Stern layer can even overcompensate for the surface charge and inverse its sign, a phenomenon known as overcharging.^114^ While the exact influence is yet unknown, the exact binding mechanism and composition of the bound layer of the surface charge is expected to have a substantial influence on slide electrification.

#### 4.3.1 Hydrodynamic slip

Hydrophobic surfaces can exhibit slip, a nonzero fluid velocity directly at the surface. It is quantified by a slip length.^115^

It is not yet clear at which length scale and if at all slip occurs at hydrophobic solid–liquid interfaces.^116,117^ Slip near the contact line would change dynamic wetting. It would also decrease the upward flow near the contact line and the stretching of the diffuse layer, see Section 1.2. These two effects would most likely influence the transfer of charge.

### 4.4 Ambient conditions

Humidity has little effect on charge separation and accumulation, at least in the range of up to 70%. At 80% relative humidity, charge separation tends to decrease.^19,43^ One reason for the weak dependence on humidity may be that a water drop creates its own humid atmosphere around it.

The presence of ions in the surrounding gas phase, *e.g.*, due to cosmic radiation,^42,118^ can contribute to charge neutralization. However, under atmospheric conditions, the ion concentration is too low to influence slide electrification substantially. Only when the ion concentration is artificially increased, *e.g.*, through the use of an ionizing air blower, ions in air reduce the surface charge and lead to neutralization.^32,89^

## 5 Open questions

The overview given in the preceding sections summarizes much of our current understanding of slide electrification. Still, many open questions remain, some of which are listed in the following.

### 5.1 Formation of interfacial charge at the hydrophob–water interface

How is the surface charge *σ*_SL_ formed at the interface between a solid hydrophobic surface and water? Is the enrichment of hydroxyl ions the dominating effect?

### 5.2 Nature of charge carriers

The formation of electric double layers at solid–liquid interfaces is well-studied. Here, we presented an overview of the quantitative evidence supporting the charge transfer mechanism based on the electric double layer structure and the factors influencing it. Apart from that, it was suggested that the transfer of electrons between the solid and the liquid plays a major role.^119^ However, for electron transfer, a quantitative comparison between experiments and a detailed physical model is largely missing. Some steps in that direction were taken by discriminating electrons from ions *via* studying the decay of the deposited charge at elevated temperatures^33^ and by assessing the influence of the work function of different materials on charge transfer.^120^ Nevertheless, the following question remains: Can the contribution of electron transfer during liquid slide electrification be unambiguously quantified experimentally? Can these measurements be compared with detailed theoretical models?

### 5.3 Atomistic processes while dewetting

We are missing a molecular picture of the transfer of charges to the solid surface at the receding contact line. How are charges deposited, despite the fact that the process is energetically unfavorable? Which atomistic processes influence the dewetting of charges? Is the deposition facilitated by a temporal formation of nanodroplets or hydration shells?

### 5.4 Liquid mixtures

Electrokinetics of liquid mixtures remains largely unexplored. Theoretical investigations show that complex layering leads to a non-trivial electric double layer structure.^121^ How does slide electrification take place in mixtures of solvents? Which effects emerge near the contact line?

### 5.5 Hydrophilic substrates

So far, slide electrification has only been observed on hydrophobic substrates, mainly due to the difficulty of moving drops on hydrophilic substrates. Theory suggests that charge separation should also occur on hydrophilic substrates, albeit reduced (Section 1.2). Can slide electrification be probed on hydrophilic substrates?

### 5.6 Surfactants and polyelectrolytes

The influence of surfactants and dissolved polyelectrolytes remains obscure. How do surfactants influence charge separation and the formation of hydration shells? Does slide electrification lead to enhanced deposition of charged surfactants and dissolved polyelectrolytes, like DNA, on the dewetted surface?

### 5.7 Surface degradation and chemical reactions

Can electric breakdown in the dielectric substrate or gas discharge at the contact line be probed in experiments? Do they degrade surfaces or lead to chemical reactions? If so, after how many wetting–dewetting cycles?

## 6 Conclusions

Slide electrification is a universal and robust phenomenon in dynamic wetting. It has been demonstrated on hydrophobic dielectric substrates with various liquid electrolytes. While most works focus on drops, it has also been reported for other dewetting scenarios. Recent experimental and theoretical findings have revealed the underlying mechanisms, parametric dependencies, and consequences of slide electrification, most notably its effect on contact angle hysteresis. Despite these advances, slide electrification still cannot be predicted with high accuracy. Atomistic processes at the contact line remain obscure. Also, the influence of slide electrification on surfaces and coatings is not yet understood. Further research is required to answer these and other open questions. Overall, slide electrification fundamentally influences dynamic wetting and must be accounted for in experiments, even when it is not their main focus.

## Author contributions

A. D. R.: investigation, writing – original draft, writing – review & editing, and visualization H.-J. B.: conceptualization, investigation, writing – original draft, writing – review & editing, and project administration S. H.: investigation, writing – original draft, and writing – review & editing S. A. L. W.: investigation, writing – original draft, and writing – review & editing.

## Data availability

No primary research results, software or code have been included and no new data were generated or analysed as part of this review.

## Conflicts of interest

There are no conflicts to declare.
